# Association Between Social Support and Perceived Stress: A Cross-Sectional Study on Staffs of the Epidemic Prevention During the Covid-19 Epidemic in China

**DOI:** 10.3389/fpubh.2022.844139

**Published:** 2022-05-20

**Authors:** Ling Zhang, Binbin Fu, Yi Xu, Qi Zhang, Shuzhen Peng, Xiaodong Tan

**Affiliations:** ^1^School of Public Health, Wuhan University, Wuhan, China; ^2^Wuhan Health Medical Cosmetic Hospital, Wuhan, China; ^3^Huangpi District People's Hospital, Wuhan, China

**Keywords:** stress perception, social support, sleep quality, psychological resilience, COVID-19

## Abstract

**Background:**

The Coronavirus Disease 2019 (COVID-19) lockdown considerably affects people's life in China, both physically and mentally. Staffs of the epidemic prevention and control in the community have played an irreplaceable role during community lockdown period in Wuhan. However, few studies have focused on their health status during epidemic prevention. This study aimed to appraise the available evidence of health conditions of them and explore the influencing factors.

**Method:**

Used a multistage sampling method, we conducted a survey in staffs of the epidemic prevention and control in the community (*N* = 503). Descriptive analysis was used to characterize the respondents. *T*-test and analysis of variance were for group differences analysis. Confirmatory factor analysis (CFA) was used to verify the scale validity, correlation analysis and pathway analysis and Structural equation model (SEM) was used to study the relationship between stress perception, social support, mental resilience and sleep quality. Statistical analysis was performed with SPSS 26.0, R version 4.1.3 and Mplus 8.3.

**Results:**

The mean Perceived Stress Scale (PSS) score of the respondents was 13.28 ± 7.31 and 51.1% had higher PSS score than the normal. In the absence of social support, people's sleeping quality and psychological resilience may decrease, their perceived stress may elevate and compromise mental health correspondingly. Social support could affect perceived stress directly, while Sleep quality and psychology resilience played significant partial mediating roles in social support affecting perceived stress. The mediating effects accounted for 50.8% of the total.

**Conclusion:**

Staffs of the epidemic prevention and control in the community suffered from poor sleep quality and high level of stress perception. Establishment of good social support may effectively reduce their stress and this effect is mediated by sleep quality and psychological resilience. Physical health status would affect the staffs' mental health and they more attention should be paid to those with poor physical health.

## Introduction

**Figure 1:**
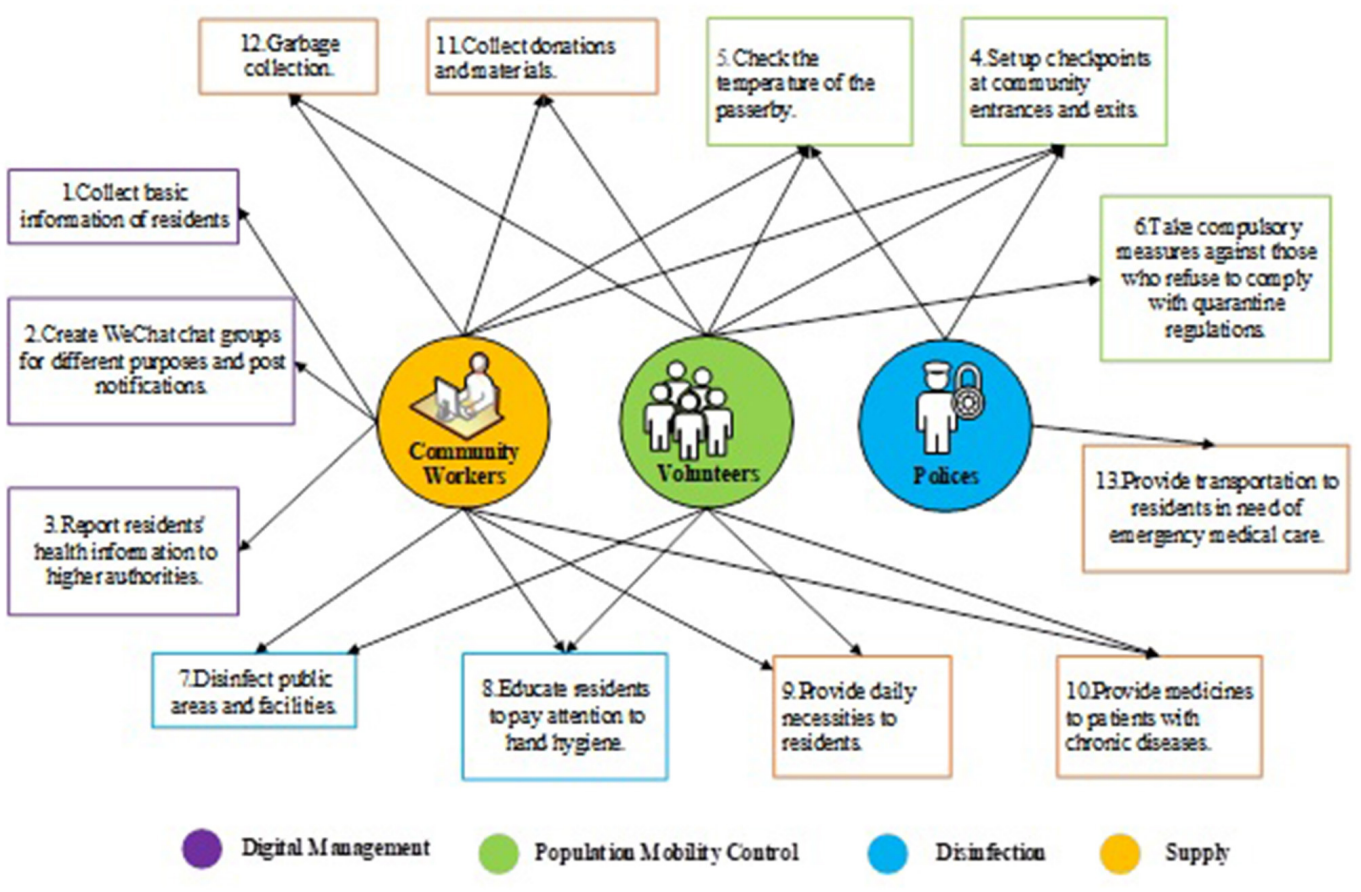
Work content of staffs of the epidemic prevention and control in the community.

COVID-19 is highly transmissible, and community lockdown strategy can effectively limit community transmission of the virus (1). In order to stop the spread of the disease, major cities in Hubei implemented lockdown strategy from January 23 to April 8, 2020. During the lockdown period, intercity travel was banned and a cordon sanitaire of Wuhan and surrounding cities in Hubei Province was established. Besides, closure and containment directives from Government included school closure, workplace closure, public transport closure, public even cancellation, restrictions on gathering and stay at home requirements. Movement restriction required residents to stay at home, only one person per household was allowed to pick up online group shopping products in the community every 1–3 days and other family members were not allowed to go out under exceptional circumstances (such as: fever, acute illness) (2). During the community lockdown period, the daily management and service of community residents are mainly taken charge of by grassroots community anti-epidemic staffs, including medical and non-medical staffs. Non-medical staffs are composed of community workers, police and volunteers. The work of non-medical staffs mainly includes collecting basic information of residents, transferring patients, disinfecting the community, and providing daily necessities to residents who are isolated at home (Figure 1) (3, 4).

The spread of COVID-19 has been proved to trigger stress, anxiety, fear, helplessness, depression and then threaten their health (5, 6) and other psychological crises grow (7) in turn. In addition to targeting physical health, we should also pay attention to people's mental health. There have been some studies on people's mental health during the epidemic, yet most of them are focused on medical staff (8) or general residents (9). Though a myriad of prevention and control measures have been employed, conditions are still grim for some countries as the virus continues to mutate and spread. Community-based prevention and control has been proved to be the backbone of the anti-epidemic system and plays an important role in maintaining efficient medical order, screening suspected patients, preventing imported cases, ensuring material support, stabilizing public sentiment, reducing disease fear, and maintaining national security (10). During the lockdown period, the cumulative number of confirmed cases in Hubei rose from <400 to more than 60,000, putting great pressure on prevention. Faced with heavy work tasks and worried that going out will infect novel coronavirus, they may suffer greater psychological pressure (11, 12). Excessive psychological pressure often leads to mental health problems, such as depression, anxiety, insomnia and so on (13–15).

Stress is reflected in the long-term interaction between people and the environment and interacts with many variables and processes. Psychological stress occurs when a person feels that the demands of the environment exceed his ability to adapt. Stress is a complex process that is constantly changing (16, 17). Everyone has different feelings toward pressure, and pressure perception is used to evaluate individual's subjective feelings toward pressure, which is defined in this study as individual's cognition and evaluation of stimulus events. By evaluating the pressure caused by stimulus events, individuals can feel whether it will threaten their internal balance. Regarding the measurement of stress, we use Perceived Stress Scale (PSS) to measure of the degree to which situations in one's life are appraised as stressful (18). Compared with pressure, pressure perception pays more attention to the individual's subjective feelings, and some scholars believe that pressure perception is more meaningful than pressure itself (19).

Previous studies have shown that there was insufficient social support and a run on medical resources in the early stage of COVID-19 epidemic, which brought great psychological pressure to the residents in Wuhan (20). The incidence of anxiety and depression of the public aged 18–76 were 26.83 and 33.46%, respectively. The average stress perception was (13.75 ± 5.22) points, which were at a moderate stress level (21). The level of stress perception of college students who study at home rose significantly during the lockdown period (22). The reported rates of stress, anxiety and depressive symptoms among medical staff in Wuhan Tongji Hospital were 29.8, 24.1, and 13.5%, respectively (23). It is not difficult to find that the currently available studies mainly focus on medical personnel and residents while few reports on non-medical staffs of the epidemic prevention and control in the community in Wuhan during the lockdown period. It is nevertheless also important to assess and protect the mental health of the staffs of the epidemic prevention and control in the community and free them from psychological problems. The study on the psychological status of 503 college students showed that there was a negative correlation between social support and perceived stress, and psychological resilience played a mediating role. Among males, the relationship between social support and perceived stress was almost entirely mediated by psychological resilience (24). A survey of 2806 college students showed that stress perception negatively predicted sleep quality, negative emotions played a partial mediating role, and social support had a moderating effect on this mediating effect (25).

Though there have been some studies on social support, stress perception, psychological resilience, and sleep quality, few explored the relationships among the four. COVID-19 has a high rate of mutation and the effects of COVID-19 are profound. Omicron variant (B.1.1.529) is extremely contagious and is raging in the world now. More and more staffs are involved in epidemic prevention. There is a critical need to ensure the physical and mental health of staffs of the epidemic prevention and control in the community as they are the backbone of combating spread. This study investigated the mental health conditions of non-medical staffs of the epidemic prevention and control in the community in Wuhan during the lockdown period, and explore the influencing factors, so as to provide a reliable basis for protecting their mental health. Based on the existing research, we put forward 3 research hypotheses: during the epidemic, (1) staffs of the epidemic prevention and control may suffer from poor mental health; (2) people's level of social support can affect their level of stress perception. (3) psychological resilience and sleep quality may play a mediating role in it. Our study may help to provide a scientific basis for psychological interventions and targeted training programs, so as to strengthen the mental health status in the face of the epidemic.

## Materials and Methods

### Participants and Procedure

The survey was carried out in Wuhan, China from March 16th to 24th, 2020. Before the survey, the study design was reviewed by local ethical committee and the investigation was carried out in accordance with the latest version of the Declaration of Helsinki. We use a multistage sampling approach. According to the urban zoning, three urban areas are randomly selected from the seven central urban areas in Wuhan: Wuchang District, Jiang'an District and Hongshan District. There are 467 communities in the 3 districts selected and then, respectively chose 15 communities at random from each district. Staffs of the epidemic prevention and control in the community selected were randomly selected to conduct an anonymous questionnaire survey. The whole sampling process was completed step by step by the survey select process of SAS 9.4 software, strictly following the principle of randomness. Due to the standard of epidemic prevention, electronic questionnaire was used instead of paper questionnaire. Considering the response rate and questionnaire efficiency, the sample size was enlarged by 10%, and the expected sample size was 495. The Inclusion criteria for this study were as follows: (1) The respondent had at least 1 month of work experience in epidemic prevention. (2) The respondent had no mental illness and had not been stimulated by adverse life events. (3) The respondent gave informed consent to this research and volunteered to participate. Exclusion criteria were as follows: (1) The respondent was older than 65. (2) The respondent was able to take shifts off.

A total of 503 questionnaires were sent out in this survey. After eliminating the ones with logic errors and those that did not meet the inclusion and exclusion criteria, 474 valid questionnaires were collected with a recovery rate of 94.23%.

### Instrument and Measures

The questionnaire included five aspects: demographics, Perceived Stress Scale (PSS), Multidimensional Scale of Perceived Social Support (MSPSS), Pittsburgh Sleep Quality Index (PSQI) and 10-item Connor-Davidson Resilience Scale (CD-RISC-10).

#### Demographics

On the basis of literature review, the questionnaire was designed according to the purpose of the study. Before conducting a formal survey, we would fully explain the purpose, meaning and use of the questionnaire to obtain the informed consent of the participants. In this part, we investigated the respondents' sex, age, educational level, marital status, work experience, contact with patients with confirmed or suspected COVID-19, height and weight. Health-related factors include chronic disease and illness within 2 weeks. Chronic diseases require participants to answer “yes” or “no” to the following questions, “whether you currently diagnosed with the following diseases: diabetes, hypertension, heart disease, arthritis, migraine, asthma, thyroid disease, heart disease, thrombosis, bronchitis/emphysema, osteoporosis, cancer, stomach/peptic ulcer, cerebrovascular disease, or other major physical diseases; the illness within 2 weeks was defined as the prevalence of acute illness in the past 2 weeks.

#### Perceived Stress Scale (PSS)

We use the Perceived Stress Scale (PSS) expressed on a 5-point Likert scale which asks respondents the frequency of occurrence of related situations in the past month developed by Cohen in 1983 to measure the degree of stress a person feels in his life (20). The frequency is expressed as never, almost never, sometimes, fairly often and very often are assigned 0–4 points, respectively. There are three versions of the scale, among which the simplified Chinese version of the 10-item scale has a moderate number of items, is widely used, and has been proved to have good reliability and validity in different populations (26, 27). Therefore, we adopted the 10-item Perceived Stress Scale in this study. The 10 items are divided into two dimensions: tension (items: 1, 2, 3, 6, 9, 10) and sense of loss of control (items 4, 5, 7, 8, reverse scored) (28). The Cronbach's coefficient of the scale in this study is 0.878 (Table 1), which suggested a good reliability.

**Table 1:** Measures of PSS, MSPSS, PSQI, and CD-RISC-10.

**Indicators**	**Scale**	**Mean**	**SD**	**Reliability of dimension (Cronbach's α)**	**Reliability (Cronbach's α)**
**Measures of perceived stress**					
p1 felt upset because of something that happened unexpectedly	0–4	1.39	1.02	0.920	0.878
p2 felt unable to control the important things in life	0–4	1.16	1.04		
p3 felt nervous and stressed	0–4	1.27	1.06		
p6 felt hard to cope with all the things that you need to do	0–4	1.12	1.00		
p9 felt angered because of things that happened that were outside of your control	0–4	1.15	0.93		
p10 there were too many difficulties and you could not overcome them	0–4	1.03	0.96		
p4 felt confident about your ability to handle your personal problems	0–4	1.32	1.14	0.834	
p5 felt that things were going your way	0–4	1.80	1.08		
p7 you were able to control irritations in your life	0–4	1.49	1.17		
p8 felt that you were on top of things	0–4	1.54	1.16		
**Measures of social support**					
s1 There was a special person who was around when you were in need.	1–7	5.30	1.46	—	0.965
s2 There was a special person with whom you could share your joys and sorrows.	1–7	5.42	1.34		
s3 Your family really tried to help you.	1–7	5.85	1.30		
s4 You got the emotional help and support you needed from your family.	1–7	5.93	1.26		
s5 You had a special person who was a real source of comfort to you.	1–7	5.49	1.38		
s6 Your friends really tried to help you.	1–7	5.46	1.39		
s7 You could count on your friends when things went wrong.	1–7	5.27	1.47		
s8 You could talk about your problems with your family.	1–7	5.54	1.40		
s9 You had friends with whom you could share your joys and sorrows.	1–7	5.54	1.34		
s10 There was a special person in your life who cared about your feelings.	1–7	5.40	1.41		
s11 Your family was willing to help you make decisions.	1–7	5.68	1.35		
s12 You could talk about your problems with your friends.	1–7	5.34	1.39		
**Measures of sleep quality**					
A subjective sleep quality	0–3	1.03	0.79	—	0.821
B sleep latency	0–3	1.27	0.98		
C sleep duration	0–3	1.03	0.90		
D habitual sleep efficiency	0–3	0.68	0.96		
E sleep disturbance	0–3	0.81	0.67		
F use of sleep medication	0–3	0.05	0.33		
G daytime dysfunction	0–3	1.01	0.94		
**Measures of psychological resilience**					
r1 Able to adapt to change.	0–4	2.86	1.20	—	0.947
r2 Can deal with whatever comes.	0–4	2.97	1.09		
r3 Tries to see humorous side of problems.	0–4	3.23	0.92		
r4 Coping with stress can strengthen me.	0–4	3.22	0.98		
r5 Tend to bounce back after illness or hardship.	0–4	2.98	1.02		
r6 Can achieve goals despite obstacles.	0–4	3.07	1.03		
r7 Can stay focused under pressure.	0–4	3.04	0.98		
r8 Not easily discouraged by failure.	0–4	3.01	1.20		
r9 Thinks of self as strong person.	0–4	3.20	0.98		
r10 Can handle unpleasant feelings.	0–4	2.94	1.08		

#### Multidimensional Scale of Perceived Social Support (MSPSS)

Social support refers to a series of support measures that a person receives through social relationships with other individuals, groups and community (11). We use the Multidimensional Scale of Perceived Social Support (MSPSS) expressed on a 7-point Likert scale developed by Zimet in 1988 to measure participants' social support (29). The scale consists of 12 items and higher scores indicating higher levels of social support. The accumulative score of each response was calculated as the total MSPSS score and a total MSPSS score <50 indicates a poor perceived social support (30). Studies have shown that the Chinese version of the scale has good reliability and validity (31). The Cronbach's coefficient of the scale in this study is 0.965 (Table 1), which suggested a good reliability.

#### Pittsburgh Sleep Quality Index (PSQI)

Sleep quality is a comprehensive evaluation index of sleep time, sleep speed, deep sleep degree and other factors. We use Pittsburgh Sleep Quality Index (PSQI) developed by Buysse in 1989 to measure sleep quality (32). The PSQI scale consists of 19 items, which are divided into seven dimensions: subjective sleep quality, sleep latency, sleep duration, habitual sleep efficiency, sleep disturbances, use of sleeping medication and daytime dysfunction. A higher score indicates poorer sleep quality. The Chinese version of the scale has good reliability and validity (33). The Cronbach's coefficient of the scale in this study is 0.821 (Table 1), which suggested a good reliability.

#### 10-Item Connor-Davidson Resilience Scale (CD-RISC-10)

Werner defines resilience as “the ability of an individual to withstand high levels of disruptive change while displaying as few undesirable behaviors as possible (34).” 10-item Connor-Davidson Resilience Scale (CD-RISC-10), which was adapted by Campbell-Stlls in 2007, was used to measure the psychological resilience of participants (35). The scale is expressed on a 5-point Likert scale and has a total of 10 items. Higher score indicates better psychological resilience (36). The Cronbach's coefficient of the scale in this study is 0.947 (Table 1), which suggested a good reliability.

### Statistical Analysis

Descriptive analysis (means ± standard errors) was used to characterize the respondents. *T*-test and analysis of variance (ANOVA) were used for the group differences analysis. The above statistical analyses were performed using SPSS 26.0. Confirmatory factor analysis (CFA) was used to verify the validity of the scales, correlation analysis, pathway analysis and structural equation model (SEM) was used to study the relationship between different scales. The maximum likelihood estimation (ML) method was used to estimate the minimum fitting criterion: χ^2^/*df* < 3, CFI ≥0.90, TLI ≥0.90, RMSEA ≤0.06 (37, 38). Mplus8.3 was used to conduct CFA, correlation analysis of latent variables, pathway analysis of scales (total score) and SEM. Pearson's correlation coefficient was calculated to test the statistical correlations between different scale (total score) via R version 4.1.3. The figures were developed using R version 4.1.3, Mplus8.3 and Office Visio 2018. *P* < 0.05 meant the difference was statistically significant. All statistical tests were two-tailed.

### Quality Control

In this study, the questionnaire was adapted from four standard scales that have been proved to have good reliability and validity and was conducted through consulting a large number of literatures and repeated revision after expert consultation. The unified instruction at the beginning of the questionnaire explains the purpose of the study and the notes for filling in to prevent bias. All the items are set as compulsory questions, and the missing items are automatically detected to ensure the integrity of the questionnaire. Recycled data ischecked and sorted by three or more people.

## Results

### Demographic Characteristics of the Respondents

The results of quantitative descriptive analysis show that 63.7% of the respondents in this study were male. Most respondents were middle-aged (38.94 ± 10.18), married (68.1%) and had worked for more than 4 years (65.6%). The majority of the respondents had attained tertiary education (73.4%) while 4.0% only attained junior high school education or below. Results of univariate analysis illustrated that there were significant differences (*p* < 0.05) between scores of PSS and PSQI of respondents who had contacted with individuals infected or suspected infected with COVID-19 (PSS = 16.04 ± 8.00, PSQI = 7.65 ± 4.40, respectively) and those had not (PSS = 12.74 ± 7.06, PSQI = 5.53 ± 3.88, respectively). Respondents with chronic disease had higher scores of PSS (14.64 ± 7.81) and PSQI (7.69 ± 4.33) than those who were not (PSS = 12.80 ± 7.08, PSQI = 5.23 ± 3.68, respectively) (*p* < 0.05). Scores of all four scales of respondents who reported illness during the last 2 weeks before surveyed (PSS = 15.72 ± 7.24, MSPSS = 63.57± 13.31, PSQI = 8.10 ± 4.05, CD-RISC-10 = 28.06 ± 8.32, respectively) were higher than those who did not (*p* < 0.05). Differences in scores of CD-RISC-10 were found between sexes. Female got lower score of CD-RISC-10 (28.90 ± 7.69) than male (31.45 ± 8.93). Refer to Table 2 for more information.

**Table 2:** Demographics of the respondents.

**Characteristics**	***n***	**Mean (±SD)**	**PSS**	**MSPSS**	**PSQI**	**CD-RISC-10**
**Age (18–65)**	474	38.94 ± 10.18				
**Hight (cm)**	150–188	169.35 ± 7.18				
**Weight (kg)**	42–120	67.65 ± 13.68				
**Gender**						
Male	302		12.85 ± 7.45	66.98 ± 14.47	5.92 ± 4.21	**31.45 ± 8.93**
Female	172		14.02 ± 7.02	64.90 ± 12.96	5.79 ± 3.63	**28.90 ± 7.69**
**Marital status**						
Married	323		12.92 ± 7.18	**67.22 ± 12.61**	6.00 ± 3.88	30.51 ± 8.30
Unmarried	151		14.05 ± 7.55	**64.09 ± 16.34**	5.61 ± 4.27	30.55 ± 9.16
**Education**						
Middle school and below	19		**15.21 ± 6.08**	53.21 ± 23.18	**4.95 ± 3.84**	23.05 ± 13.17
Senior school	107		**10.95 ± 7.29**	67.25 ± 14.74	**4.49 ± 3.85**	31.93 ± 9.74
College and above	348		**13.89 ± 7.24**	66.62 ± 12.72	**6.35 ± 3.96**	30.56 ± 7.67
**Job tenures**						
<1year	51		11.94 ± 6.80	66.76 ± 14.15	**5.51 ± 4.42**	31.92 ± 8.01
1–3 years	112		13.41 ± 7.69	66.00 ± 15.26	**5.67 ± 4.51**	29.96 ± 9.67
4–6years	80		12.54 ± 7.13	64.65 ± 12.71	**5.44 ± 3.65**	30.46 ± 8.51
7–10years	63		12.81 ± 5.22	65.59 ± 13.91	**4.79 ± 3.05**	30.06 ± 7.12
>10years	168		14.13 ± 7.91	67.20 ± 13.67	**6.73 ± 3.87**	30.68 ± 8.54
**Contact with individuals infected** **for suspected infected with COVID-19**						
Yes	77		**16.04 ± 8.00**	65.40 ± 14.93	**7.65 ± 4.40**	30.26 ± 8.39
No	397		**12.74 ± 7.06**	66.38 ± 13.78	**5.53 ± 3.88**	30.57 ± 8.62
**Prevalence of chronic disease**						
Yes	124		**14.64 ± 7.81**	65.19 ± 13.82	**7.69 ± 4.33**	29.37 ± 8.47
No	350		**12.80 ± 7.08**	66.59 ± 14.02	**5.23 ± 3.68**	30.93 ± 8.59
**Two-week prevalence**						
Yes	140		**15.72 ± 7.24**	**63.57 ± 13.31**	**8.10 ± 4.05**	**28.06 ± 8.32**
No	334		**12.25 ± 7.10**	**67.34 ± 14.10**	**4.94 ± 3.61**	**31.56 ± 8.48**

*Bold font indicates the presence of significant differences among groups (p < 0.05)*.

### Status of Individuals During the Epidemic

PSS, MSPSS, PSQI and CD-RISC-10 were applied to measure the status of individuals' perceived stress, social support, sleep quality and psychological resilience, and the mean scores were 13.28 ± 7.31, 66.22 ± 13.97, 5.87 ± 4.01, 30.52 ± 8.58, respectively. The mean PSS score of the respondents was higher than the normal level and 51.1% had a PSS score >13, suggesting a high level of people's perceived stress during the epidemic, which may lead to some psychological problems. 46.2% respondents had a PSQI score >5, indicating poor status of sleep quality. 16.2% respondents reported a poor perceived social support. We further compared the conditions of perceived stress and social support in different populations. Results from independent sample *t*-test illustrated that the conditions of social support and perceived stress in better-educated staffs (staffs who had acquired tertiary education) was better than that of better-educated (staffs who had not acquired tertiary education). Staffs who had been confirmed or suspected to be infected with COVID-19 were in worse status of social support and perceived stress than staffs who were not. Moreover, our results revealed that marriage showed a protective effect on staffs' social support and perceived stress.

**Figure 2:**
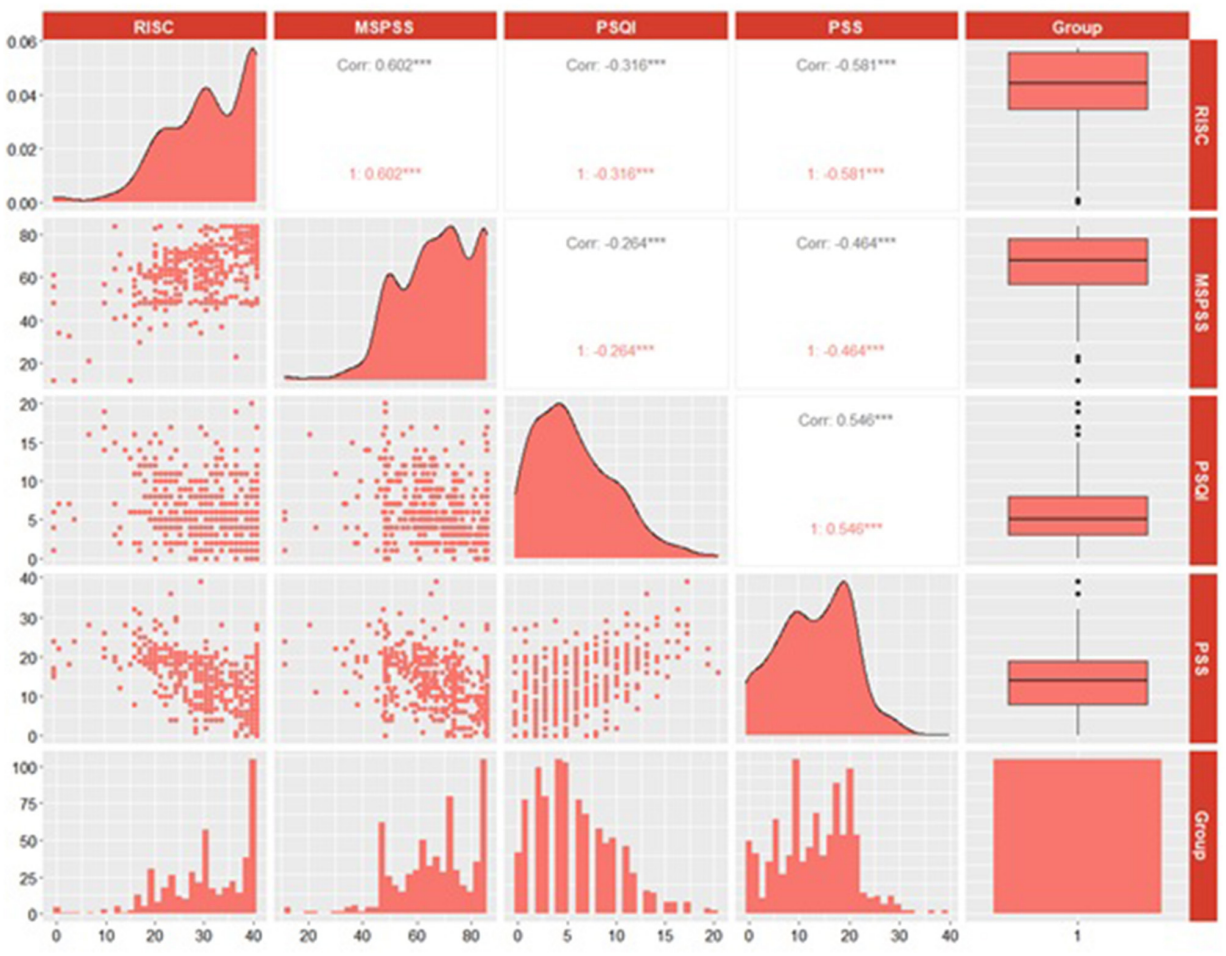
Correlation analysis of PSS, MSPSS, PSQI, and CD-RISC-10.

**Figure 3:**
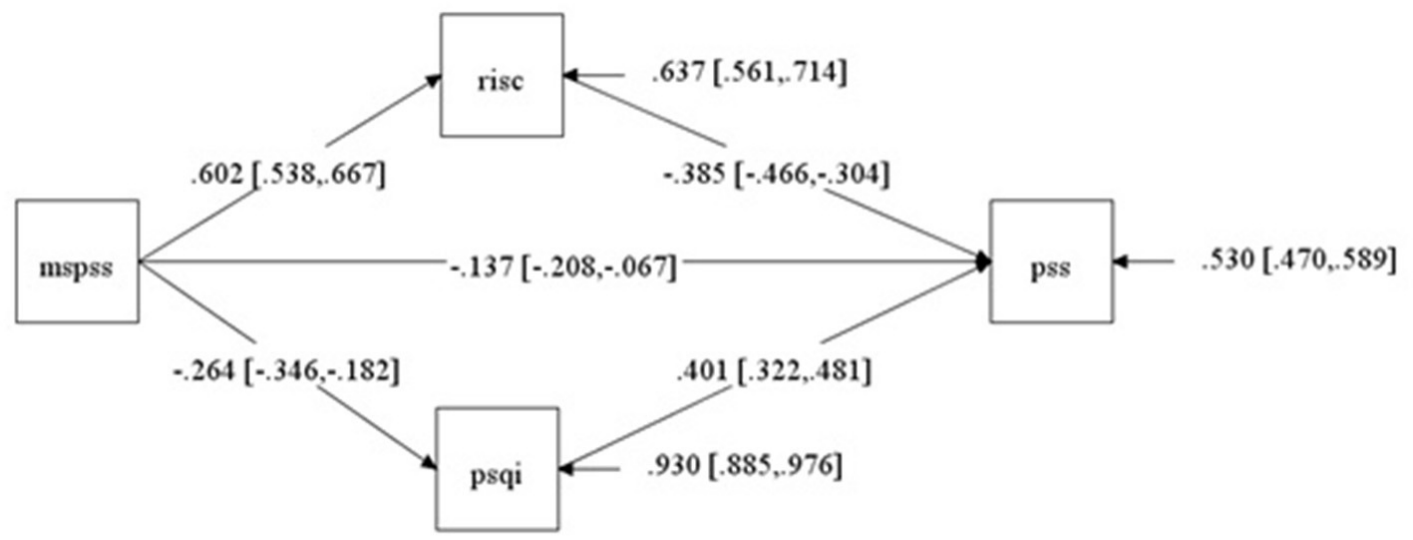
Path analysis of PSS, MSPSS, PSQI, and CD-RISC-10.

Correlation analysis and pathway analysis were conducted to explore the relationship between different scales. Result of correlation analysis showed that there were significant correlations (Figure 2) between MSPSS, CD-RISC-10, PSQI and PSS (*p* < 0.001). MSPSS was positively correlated with CD-RISC-10 (*r* = 0.602, *p* < 0.001), while negatively correlated with PSQI (*r* = −0.264, *p* < 0.001) and PSS (*r* = −0.464, *p* < 0.001). PSQI and PSS were negatively correlated with CD-RISC-10 (*r* = −0.316, *p* < 0.001; *r* = −0.581, *p* < 0.001). PSQI was positively correlated with PSS (*r* = 0.546, *p* < 0.001). Based on the correlation analysis results, we conducted a path analysis and similar relationships between each scale were obtained. Besides, significant mediations of CD-RISC-10 and PSQI in MSPSS affecting PSS were found (Figure 3). The indirect effects of CD-RISC-10 and PSQI were −0.232 (*p* < 0.001) and −0.106 (*p* < 0.001), accounting for 48.84 and 22.32% of the total effect, respectively (Table 3).

**Table 3:** Path analysis of social support, stress perception, sleep quality, and psychological resilience of respondents.

**Path**	**Direct effect**	**Indirect effect**	**Effect ratio**
MSPSS → PSS	−0.137^***^		28.84%
MSPSS → CD-RISC	0.602^***^		
CD-RISC → PSS	−0.385^***^		
MSPSS → CD-RISC → PSS		−0.232^***^	48.84%
MSPSS → PSQI	−0.264^***^		
PSQI → PSS	0.401^***^		
MSPSS → PSQI → PSS		−0.106^***^	22.32%
Total effect		−0.475^***^	

*** *p < 0.001*.

### Goodness of Fit of CFA and SEM

The confirmatory factor analysis (CFA) was applied to assess the construct validity of the scale. Significant correlations of latent variables were observed through CFA (*p* < 0.001). Social support was positively correlated with psychological resilience (*r* = 0.623, *p* < 0.001) and perceived stress (*r* = 0.816, *p* < 0.001). Psychological resilience was positively correlated with perceived stress (*r* = 0.639, *p* < 0.001).

The structural equation modeling (SEM) was used to provide an interpretative modeling structure that accounted for the multivariate relationships between variables in the models. In order to make the results easier for readers to understand, measurement of sleep quality was back-transformed for presentation and the calculation formula was as follows: *sleep* < *uscore* > *quality* = 21 − *PSQI*. Higher score indicated better sleep quality. Two models were performed (Model 1 & Model 2) to examine the effect of social support on perceived stress and the mediating effects of psychological resilience and sleep quality. Based on Model 1, a chained mediating model was applied in Model 2: social support → sleep quality → psychological resilience → perceived stress. In the mediating analysis, bootstrap method (bootstrap = 500) was used. Results of Model2 showed that the chain mediation effect did not exist, so we chose the results of Model1 as the final model. The fit statistics showed a good model fit (Table 4).

**Table 4:** Goodness of fit of CFA and SEM.

**Indicator**	**Criteria**	**CFA**	**SEM**
χ^2^	-	1093.852	1313.267
*df*	-	449	482
χ^2^/*df*	<3	2.436	2.725
RMSEA	≤0.06	0.055	0.060
CFI	≥0.90	0.951	0.938
TLI	≥0.90	0.946	0.932

### The Mediating Role of Psychological Resilience and Sleep Quality in Social Support Affecting Perceived Stress

**Figure 4:**
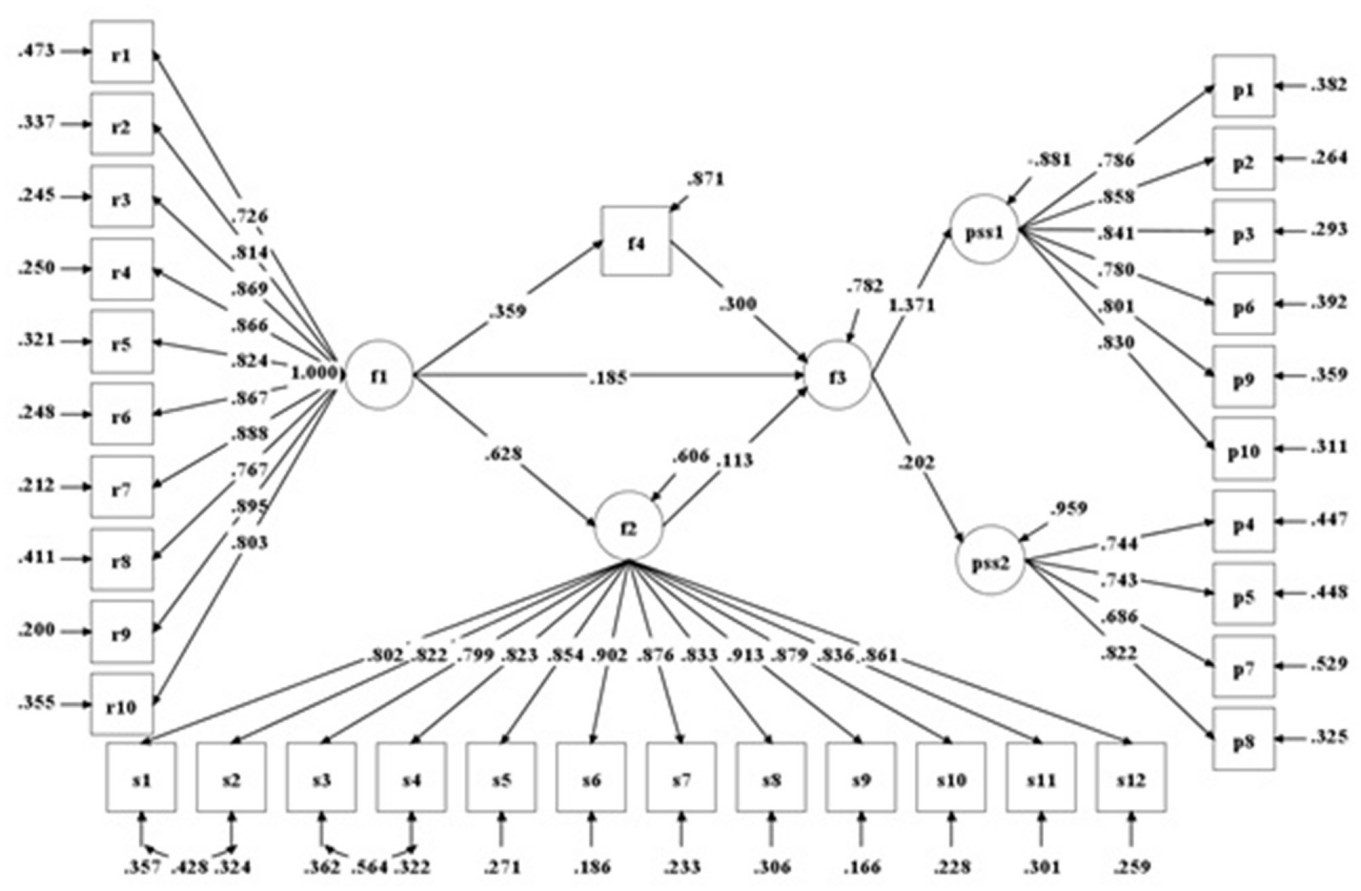
Structural equation model of social support and perceived stress (standardized coefficients in Model 1) (all path coefficients are significant, *p* < 0.001).

Model 1 was ultimately selected as the best model. Figure 4 shows the standardized path coefficients of direct and mediating effects in Model 1. As expected from the results, there were direct positive relationships between the respondents' perceived stress and their social support (*b* = −0.179, *p* < 0.001), psychological resilience (*b* = −0.119, *p* < 0.001) as well as their sleep quality (*b* = −0.304, *p* < 0.001) significantly. The respondents' social support was positively correlated with their psychological resilience (*b* = 0.634, *p* < 0.001) and sleep quality (*b* = 0.363, *p* < 0.001). Both psychological resilience and sleep quality played a partial mediating role in social support affecting perceived stress. The overall proportion explained by the mediating effect of psychological resilience and sleep quality was 50.8% (20.6 and 30.2%, respectively). The mediating effect of sleep quality (*b* = 0.110, *p* < 0.001) was stronger than that of psychological resilience (*b* = 0.075, *p* < 0.001).

## Discussion

Based on a multistage sampling cross-sectional study conducted from March 16 to March 24, 2020, we appraised the available evidence of health conditions of community epidemic prevention staffs in Wuhan and explored the influencing factors. Similar to previous studies (39), we found that non-medical community epidemic prevention staffs were in poor conditions of psychological status and sleep quality. It was observed that most respondents (51.1%) had high level of perceived stress, 16.2% respondents perceived a poor social support and 46.2% had poor quality of sleep during the epidemic prevention of COVID-19. Differences in four scales surveyed between male and female were not found expect for CD-RISC-10 in the presented study. Female showed weaker psychological resilience than male. Physical health status would affect the staffs' mental health. Specifically, respondents with chronic disease or respondents who had contacted with individuals infected or suspected infected with COVID-19 perceived higher stress and had worse quality of sleep than those not. Respondents who reported illness during the last 2 weeks before surveyed perceived higher stress, lower level of perceived social support, worse quality of sleep and weaker psychological resilience. It was also found that elevated social support would help reduce perceived stress of staffs of the epidemic prevention and control in the community, and both psychological resilience and sleep quality played important mediating roles in the process of social support affecting perceived stress.

Community lockdown during the epidemic is a very common measure, we should pay attention to people's health conditions not only the isolated people' but also the executors'. The staffs of the epidemic prevention and control are important personnel on the front lines in their departments and perhaps experience more safety problems and suffer from more stress. As the front line of epidemic prevention and control, non-medical community epidemic prevention staffs bore more risk and greater stress during the epidemic and were more likely to suffer from anxiety and depression than medical staffs (39). Attention should be paid to their mental health. Although as a special professional group, they have experienced psychological training, high-intensity continuous work and strong psychological load under the background of sudden epidemic will still bring them greater problems physically and mentally.

According to our study, more scientific and targeted management measures should be taken by relevant management department to tackle the unsatisfying mental health conditions and sleep quality of the community epidemic prevention and control staffs in the battle against COVID-19, so that they can carry out the front-line epidemic prevention work with better physical and mental state. Measures such as reasonably organizing their working hours and ensuring adequate sleep can be taken to reduce their perceived stress. An approach to obtain the optimal peak load shifting plan is probably justified. The COVID-19 pandemic could to some extent be termed as a loneliness pandemic for it requires maintaining social distance, which inhibits the development of social interaction. We need to consider some ways to keep pace with practice to improve the level of social support, for example, provide timely psychological comfort and psychological guidance for them, especially for female and staffs with poor physical health.

Though our study offers a novel angle to reduce people's perceived stress, this study may have the following limitations. On the one hand, we only use social support at the self-perception level, and there is no sufficient evidence to test the probable influence mechanisms of actual social support on perceived stress. The measurement of social support is subjective, and it is influenced by individuals' psychological state. The scale with objective social support index can be used in later research, such as the Social Support Questionnaire (SSQ) and the Interview Schedule for Social Interaction (ISSI) (40). On the other hand, when examining the mediating variables of psychological resilience and sleep quality, we actually set perceived stress as the terminal of causal path, which is based on the previous studies, but they may have a causal relationship with each other, which is subject to the inherent limitations of cross-sectional data, which needs to be further tested by tracking data.

## Conclusion

In addition to opportunistic infections, staffs of the epidemic prevention and control in the community suffered from poor sleep quality and high level of perceived stress. Establishment of good social support may help improve sleep quality, elevate personal psychological resilience and decrease perceived stress. Social support may effectively affect their stress and this effect is mediated by sleep quality and psychological resilience. Physical health status would affect the staffs' mental health and they more attention should be paid to those with poor physical health.

## Data Availability Statement

The raw data supporting the conclusions of this article will be made available by the authors, without undue reservation.

## Ethics Statement

The studies involving human participants were reviewed and approved by Ethics Committee of Wuhan University. Written informed consent for participation was not required for this study in accordance with the national legislation and the institutional requirements.

## Author Contributions

LZ: data analysis, writing—original and revised draft, and methodology. BF: data collation and writing-original draft. YX: data collection, data management, and preparation. QZ: data collection, data management, and preparation. SP: conceptualization, data curation, writing—review and editing, and validation. XT: conceptualization, data curation, writing—review and editing, validation, and supervision. All authors contributed to the article and approved the submitted version.

## Conflict of Interest

The authors declare that the research was conducted in the absence of any commercial or financial relationships that could be construed as a potential conflict of interest.

## Publisher's Note

All claims expressed in this article are solely those of the authors and do not necessarily represent those of their affiliated organizations, or those of the publisher, the editors and the reviewers. Any product that may be evaluated in this article, or claim that may be made by its manufacturer, is not guaranteed or endorsed by the publisher.
